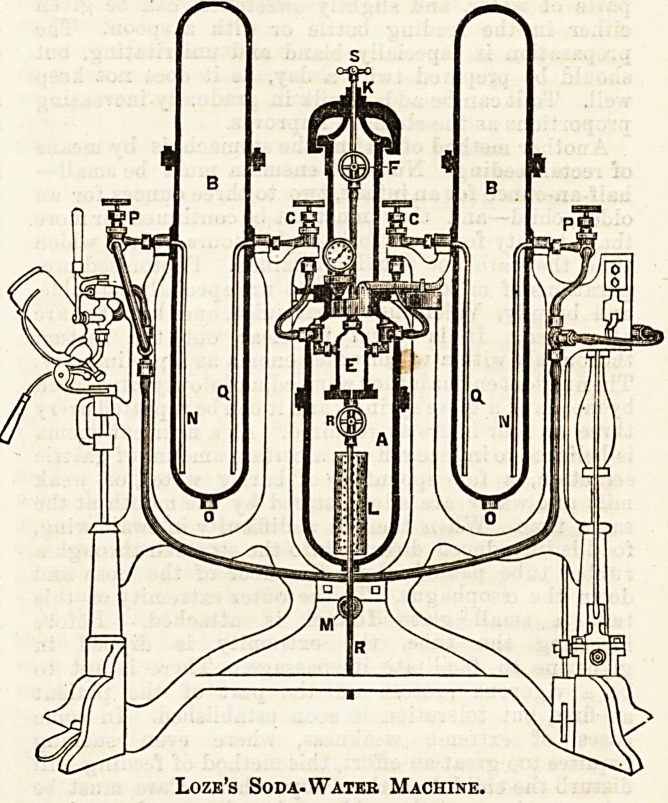# New Drugs, Appliances, and Things Medical

**Published:** 1892-11-19

**Authors:** 


### NEW DRUGS, APPLIANCES, AND THINGS MEDICAL.

[All preparations, appliances, novelties, etc., of which a notice is desired, should be sent for the Editor, to care of The Manager, 140, Strand, London, W.C.]

### A NEW FORM OF SODA-WATER MACHINE.

We have received from Lozé and Co., Liverpool, plans and descriptions of their improved forms of machines for the manufacture of aerated waters. Most machines for this purpose are made for the use of chalk in some form. As the acid used to liberate the carbonic acid is sulphuric, sulphate of lime, or plaster of Paris is the waste material formed. Now, this process has several drawbacks. The carbonic acid has to be well washed before it can be used for aerating purposes, as it contains unpleasant smelling materials which

Loze's Soda-Water Machine.

would spoil any soda or other sparkling water. Again, the plaster of Paris waste is practically useless—very bulky and difficult to get rid both from the machine and afterwards. Messrs. Lozé and Co. use soda bicarbonate in their machines. This process has the following advantages: 1. The gas given off is pure, and requires no washing, and so can be led into the fluid to be aerated at once. 2. The soda compound yields about three times as much gas as chalk does. 3. The resulting sulphate of soda has a marketable value as Glauber's salts. In examining the plans of the machines we were much impressed with the following: 1. Compactness. 2. Ease of working. 3. Facility of cleaning and replacing worn or broken parts. 4. Continuous gas pressure. 5. And not the least, reasonable cost. We publish a block with description of the machines, which vary in size from one suitable for a retail chemist or an hotel to those required by the largest wholesale firms. We also note the manufacturers make every requisite for mineral water producers.

The Royal Perfumery Company, Bond Street.—In our notice of the specialities of the above company, we inadvertently omitted to give the full name of the owners, Messrs. Napoleon Price. Both the cucumber and glycerine and velveen soaps supplied by them are so excellent, we should be sorry to deprive Messrs. Napoleon Price of the credit due to them.

## Figures and Tables

**Figure f1:**